# Active production and passive observation of hand movements shift visual hand location

**DOI:** 10.1038/s41598-023-47557-z

**Published:** 2023-11-24

**Authors:** Maryvonne Langenberg, Manuel Bayer, Eckart Zimmermann

**Affiliations:** Institute for Experimental Psychology, Heinrich Heine University Düsseldorf, Universitätsstr. 1, 40225 Düsseldorf, Germany

**Keywords:** Human behaviour, Motor control, Sensorimotor processing

## Abstract

Which factors influence the perception of our hand location is a matter of current debate. Here, we test if sensorimotor processing contributes to the perception of hand location. We developed a novel visuomotor adaptation procedure to measure whether actively performing hand movements or passively observing them, influences visual perception of hand location. Participants had to point with a handheld controller to a briefly presented visual target. When they reached the remembered position of the target, the controller presented a tactile buzz. In adaptation trials, the tactile buzz was presented when the hand had not yet reached the target. Over the course of trials, participants adapted to the manipulation and pointed to a location between the visual target and the tactile buzz. We measured the perceived location of the hand by flashing a virtual pair of left and right hands before and after adaptation. Participants had to judge which hand they perceived closer to their body on the fronto-parallel plane. After adaptation, they judged the right hand, that corresponded to the hand used during adaptation, to be located further away from the body. We conclude that sensorimotor prediction of the consequences of hand movements shape sensory processing of hand location.

## Introduction

How do we know where our hands are located in space? Sensory signals like vision and proprioception report the position of the hand. These signals need to be integrated in order to provide a final estimate of hand location. To study the mechanisms of integration, adaptation experiments apply an artificial spatial discrepancy between vision and proprioception^1^. The first and most famous example of visuomotor adaptation is prism adaptation^2^. Wearing prismatic goggles that displace vision to a certain amount leads, when the goggles are taken off, to adaptive aftereffects such that subjects misreach toward objects^3^. In other words, after a repeated exposure to the discrepancy, the hand of the participant feels to be located closer to the false visual feedback. The aim of visuomotor adaptation is to minimize the error between the terminal location of a hand movement and the target object. End-point errors in pointing or reaching tasks might derive from the target having been displaced during movement execution or from execution noise leading to an inaccurate movement. Separate mechanisms control the error processing of both signals^4^. Human imaging studies have mostly reported that cortical error processing occurs in parietal areas^4,5^. However, a monkey study has provided evidence that motor and premotor cortices encode information on end-point errors in reaching^6^. These signals might install changes in the cerebellum which is indispensable for successful adaptation^7–12^.

However, the discrepancy between vision and proprioception in reaching end-point errors could also be resolved by shifting the visual signal of the hand position toward the proprioceptive signal. Studies investigating active hand movements have addressed this question by asking subjects to judge the position of a cursor after they had been adapted to a visuomotor rotation^13,14^. A shift of the cursor toward the felt position of the hand was indeed observed. However, the visual shift remained smaller than the proprioceptive. The standard explanation for the stronger shift in proprioception is usually explained along the lines of optimal integration. In this view, vision is the dominant sense with regard to space, since it is least variable. If the visual and the proprioceptive signals are integrated in an optimal fashion, each would be weighted by their own uncertainty^15,16^. This bayesian principle predicts a shift of the more variable sense towards the more precise as is found in the proprioceptive drift. However, in contrast to this explanation, studies have reported the absence of a correlation between the proprioceptive shift and proprioceptive variability which should be expected if the bayesian model would explain visuomotor adaptation^17^.

An alternative explanation for the stronger shift in proprioception compared to vision might be the experimental construction of the artificial error. In visuomotor adaptation studies, the position of the visual signal representing the location of the hand, for instance a cursor, is distorted with respect to the physical location of the hand. In the first adaptation trials participants will see that a movement which brought the cursor to the target before the distortion was applied, now leaves the cursor far away from the target object. Compensating for the visual displacement will minimize the visual error and create a proprioceptive error. This latter error can only be minimized internally by calibrating perception. Finding proprioceptive adaptation aftereffects therefore might be the consequence of how the error signal was presented. With virtual reality methods, it is possible to provide wrong tactile feedback about movement success. Participants can be tricked into believing that, according to their proprioceptive sense, they already reached the target although visually they seem not yet to have reached it. In this scenario, stronger visual aftereffects might be found, because it is the visual modality that is forced to compensate for the discrepancy between the senses. In the theory of active inference that shares assumptions about sensorimotor processing, motor actions are conceived of as an experiment that test our assumptions about the external world^18,19^. A proprioceptive prediction error would observed at movement termination would falsify the internal belied about the sensory consequences of movement execution. The sensorimotor system therefore must update the belief by adapting and—if perceived hand location relies on sensorimotor processes—also update the internal representation of hand location.

**Figure 1:**
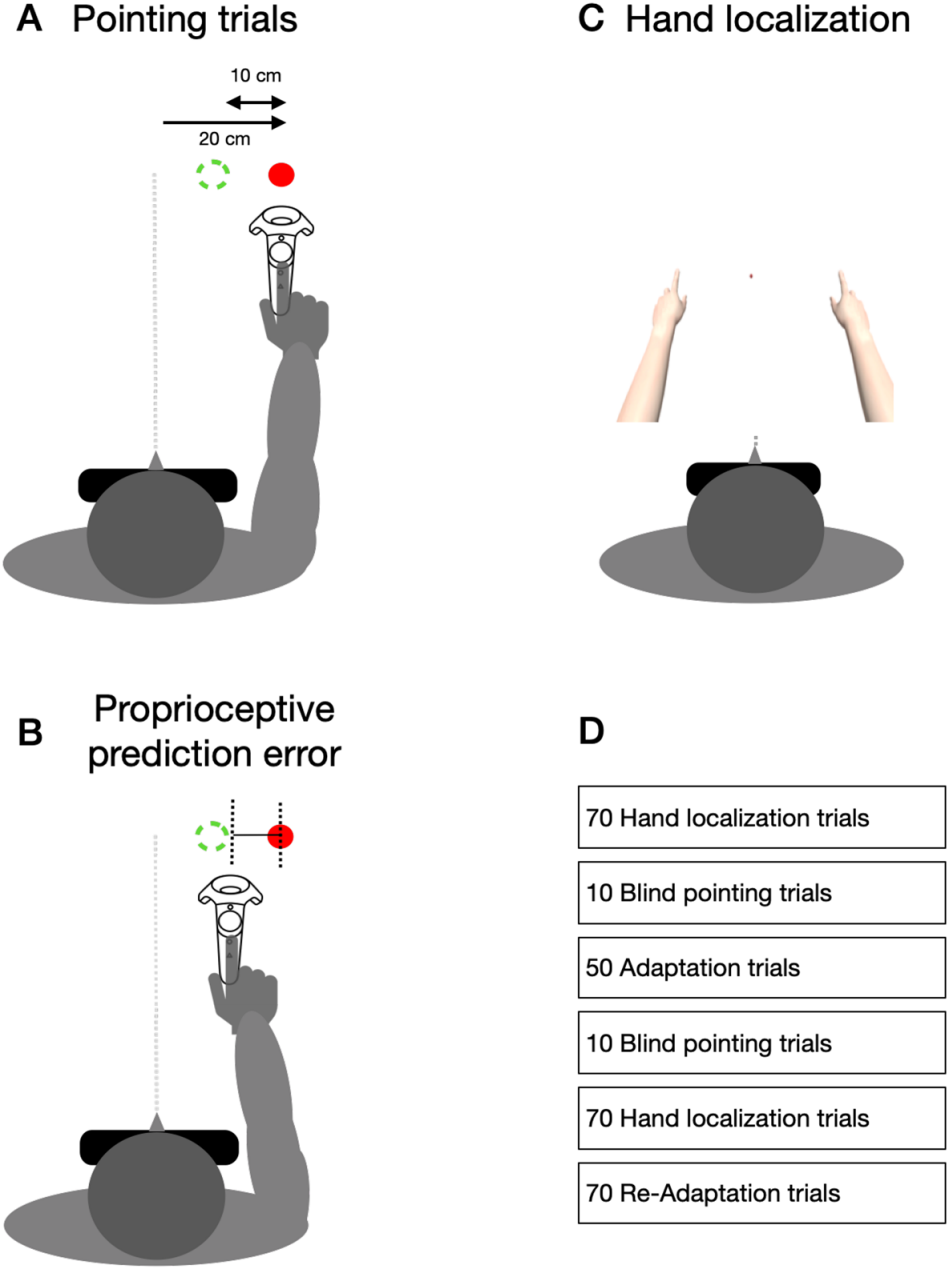
(**A**) Illustration of the setup used in pointing trials, showing the movement start and the movement end position. Subjects directed their gaze toward a fixation point presented in the head-mounted display in their central field of view. They used a handheld controller to point to a visual target that was briefly presented 40 cm in front and 20 cm to the right of the midsagittal plane. In pre-adaptation pointing trials, a tactile buzz was presented by the controller when the hand landed on the target location. In adaptation trials, the buzz was presented before the hand reached the target location. The spatial discrepancy between the time of buzz presentation and the visual target location increased gradually until it reached a distance of 10 cm in horizontal direction. (**B**) Illustration of the proprioceptive prediction error, induced by the adaptation procedure. By presenting spatially distorted tactile feedback about movement success, an error between the predicted proprioceptive position of the target and the indicated location by the experimental feedback is produced. (**C**) Illustration of hand localization trials. Participants placed their physical hands on the table in an outstretched position. In the virtual environment, the virtual hands were flashed for 100 ms simultaneously on each body side. The right hand, i.e. the probe hand was always presented at the same location, i.e. the location in the virtual environment that matched the physical position of the hand. The position of the left hand, i.e. the reference hand was varied across trials in order to measure the perceived position of the right virtual hand. Participants were required to respond which hand appeared closer to the midsagittal plane. (**D**) Trial-structure of the experiment.

To investigate if visual aftereffects can follow visuomotor adaptation, in the present study, we created a discrepancy between the predicted proprioceptive position when reaching the target and the actual proprioceptive position when the tactile feedback was provided (see Fig. 1A,B). Participants received a buzz on their index finger when they pointed toward a briefly flashed visual target that was presented in a head-mounted display. In baseline trials, participants received the buzz when their finger position corresponded to the position where the target had been shown. During adaptation, the buzz was presented shortly before the hand reached the target. The spatial displacement gradually increased across trials until it reached 10 cm. In separate sessions, we also varied the certainty of the visual hand. We either showed the visual hand for the entire movement or we flashed it only briefly during the course of the movement or we did not show it at all. In order to estimate the perceived visual hand location, we asked participants before and after adaptation to compare the visual location of virtual hands flashed in the left and in the right periphery.

## Results

### Experiment 1: active pointing with perturbation

**Figure 2:**
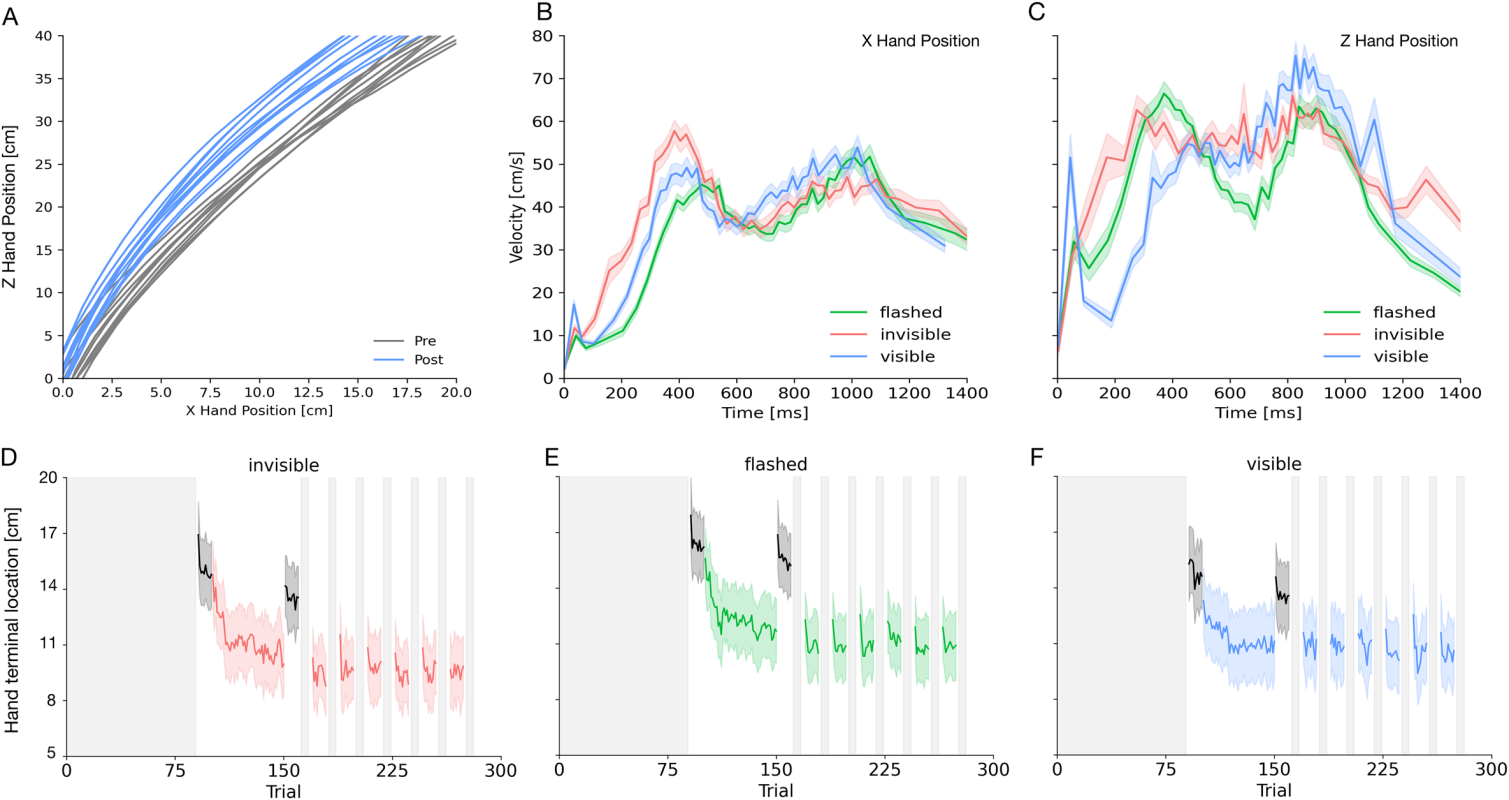
(**A**) Example pointing movement trajectories for one subject from a session in which the virtual hand was visible for the depth (Z Hand Position) and the horizontal (X Hand Position) location. (**B**) Average velocity profile of pointing trajectories (X Hand Position) for the flashed (shown in green), the invisible (shown in red) and the visible condition (shown in blue). The thick lines represent the mean across all subjects and the shaded area the standard error of the sample mean. (**C**) Average velocity profile of pointing trajectories (Z Hand Position). Same conventions as in Figure (**B**). (**D–F**) Hand terminal x-components of the pointing movement across trials for the invisible, the flashed and the visible condition. Pointing data from blind pointing trials are shown in gray and data from adaptation trials in color (same conventions as in Figure (**B**)).

In Experiment 1, subjects were required to point to a visual target that was briefly flashed (see Fig. 1A,B). As soon as they reached the target position, tactile feedback was provided via a buzz in the hand-held controller. In adaptation trials, the buzz was presented before the hand had arrived at the target location. The distance between the target location and the position, where the buzz was provided, increased over 10 trials until it reached 10 cm. Figure 2D–F shows the time course of the pointing terminal positions averaged across participants. In the first trials, participants pointed close to the veridical location of the visual target. However, as soon as the presentation of the tactile feedback was shifted away from the visual target position, participants adjusted their pointing behavior gradually across trials and pointed closer to the position at which the tactile feedback was presented.

In order to test aftereffects of pointing adaptation, before and after adaptation, we measured pointing performance in blind pointing trials. In these trials, no tactile feedback about movement success was provided. Pointing locations averaged across subjects are shown in Fig. 2 as black dots. One can see that in the pre-adaptation blind pointing trials, participants landed close to the veridical position of the visual target. However, in blind pointing trials measured after adaptation, pointing was shifted in direction where the tactile feedback was presented in the adaptation trials. For each participant, we selected the blind pointing trials measured before and after adaptation (each 10 trials) and computed linear mixed models. The results of the LMM showed that participants pointed further inward, i.e. closer to their own body, in the post compared to the pre adaptation trials (β = 1.23, SE = 0.23, t = 5.36, p < 0.001). They also pointed further in the flashed compared to the visible condition (β = 1.74, SE = 0.28, t = 6.15, p < 0.001) and in the invisible compared to the visible condition (β = 0.94, SE = 0.28, t = 3.33, p < 0.001). The final pointing position was also further away from the start in the flashed compared to the invisible condition (β = 0.80, SE = 0.28, z = 2.82, p = 0.005). These results confirm that the discrepancy between the visual target position and the shifted tactile feedback induced visuomotor adaptation.

In principle, participants could have solved the pointing task by moving their hand until they received the tactile feedback and then stop immediately. In order to estimate whether subjects used this strategy or whether they performed goal-directed pointing movements, we analyzed peak velocities of the pointing trajectories. If the artificial discrepancy between the visual target location and the tactile feedback affected movement planning, peak velocities after adaptation should be lower than before. Planning smaller pointing movements should yield lower peak velocities. By contrast, if subjects just moved their hand until they received tactile feedback, no change in peak velocities would be expected. The results of the LMM showed that peak velocities were lower in the post compared to the pre adaptation trials (β = 3.20, SE = 1.11, t = 2.87, p = 0.004). Peak velocities were higher in the visible compared to the flashed condition (β = 4.64, SE = 1.36, t = 3.41, p < 0.001). There was no difference in maximum pointing speed between the invisible and visible condition (β = 2.03, SE = 1.36, t = 1.49, p = 0.136). Participants in the invisible condition had a higher maximum pointing speed than in the flashed condition (β = 6.68, SE = 1.36, z = 4.90, p < 0.001).

The sphere that indicated the home position had a comparably wide diameter, such that subjects could re-position their hand even without seeing it. In principle, subjects could have systematically shifted the start position of their movements across a session. To check for this possibility, we analyzed the start location of the subjects’ physical hands before and after adaptation and found that, according to the results of the LMM, they were statistically indistinguishable (β = − 0.02, SE = 0.02, t = − 0.92, p = 0.0.36).

**Figure 3:**
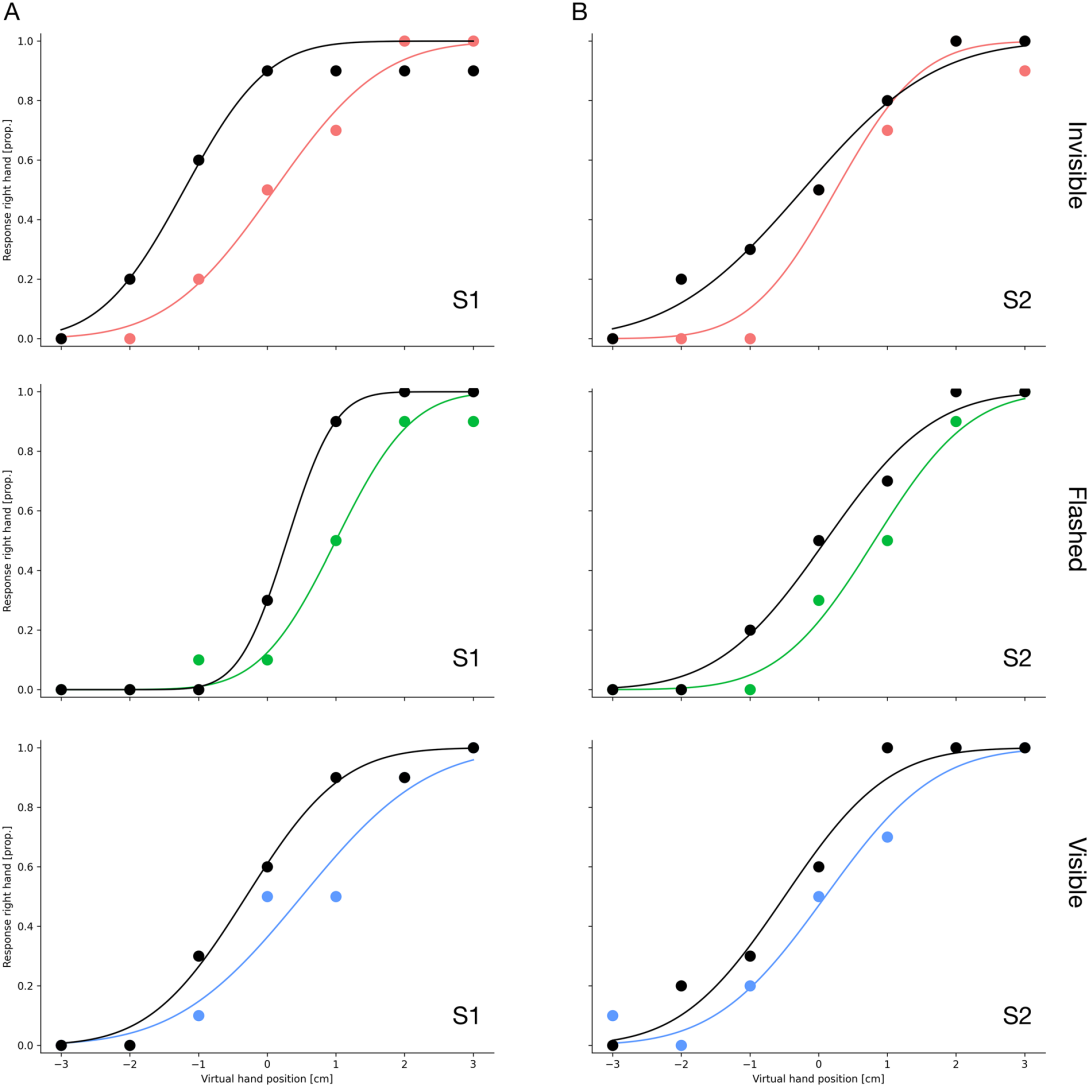
Example psychometric functions for two participants measured in the pre-adaptation (shown in black) and the post-adaptation hand localization trials (shown in color) for the invisible, the flashed and the visible condition from two participants (S1 and S2). Positive numbers indicate that the right hand was perceived as more outward than the left hand and negative numbers indicate that the right hand was perceived as closer to the body than the left hand.

We then asked how adaptation of pointing movements would affect visual perception of the hands (see Fig. 1C). To this end, we implemented hand localization trials in which subjects had to keep their gaze directed at the fixation point. Two hands were simultaneously presented on the left and right side for 100 ms. Subjects had to decide which hand appeared closer to the midsagittal plane. Figure 3 shows example psychometric functions for 2 observers from before (in black) and after adaptation (in color). Data points represent the percentage that subjects judged the right hand to be closer to the midsagittal plane than the left hand. The right hand was always presented at the same position and the left hand location varied across trials in seven equiprobable and equidistant steps. One can see that subjects were well able to perform the task. For both subjects, psychometric functions were shifted toward the right after adaptation, indicating that participants judged the right hand to be further away from the midsagittal plane than the left hand.

**Figure 4:**
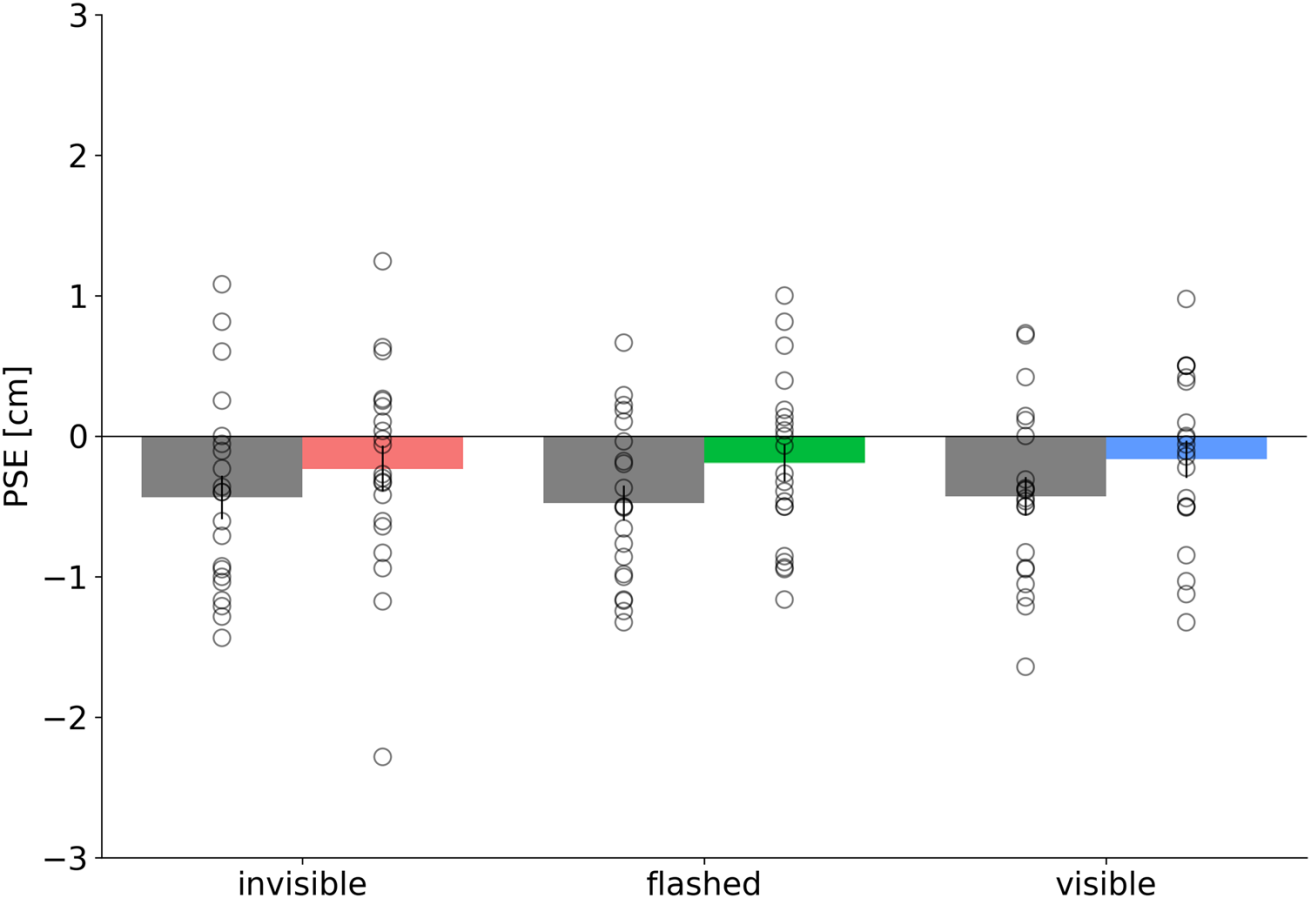
Average deviation of post-adaptation hand localization PSEs from pre-adaptation trials for the invisible, the flashed and the visible condition. PSEs refer to the point where both hands appear at an equal distance relative to the midsagittal plane. Open circles show single subjects data. Error bars represent the standard of the sample mean.

Mean hand location judgments of the right hand location are shown in Fig. 4. On average, participants estimated the right hand to be further outward after adaptation. A 2 × 3 repeated measures ANOVA with the factors adaptation (pre/post) and condition (invisible, flashed and visible) confirmed a significant main effect of the factors pre/post (F(1, 20) = 7.29, p = 0.014), indicating that pointing adaptation changed apparent hand position. The main effect condition (F(1, 20) = 0.08, p = 0.93) and the interaction effect were not significant (F(2, 40) = 0.15, p = 0.86).

### Experiment 2: active pointing without perturbation

**Figure 5:**
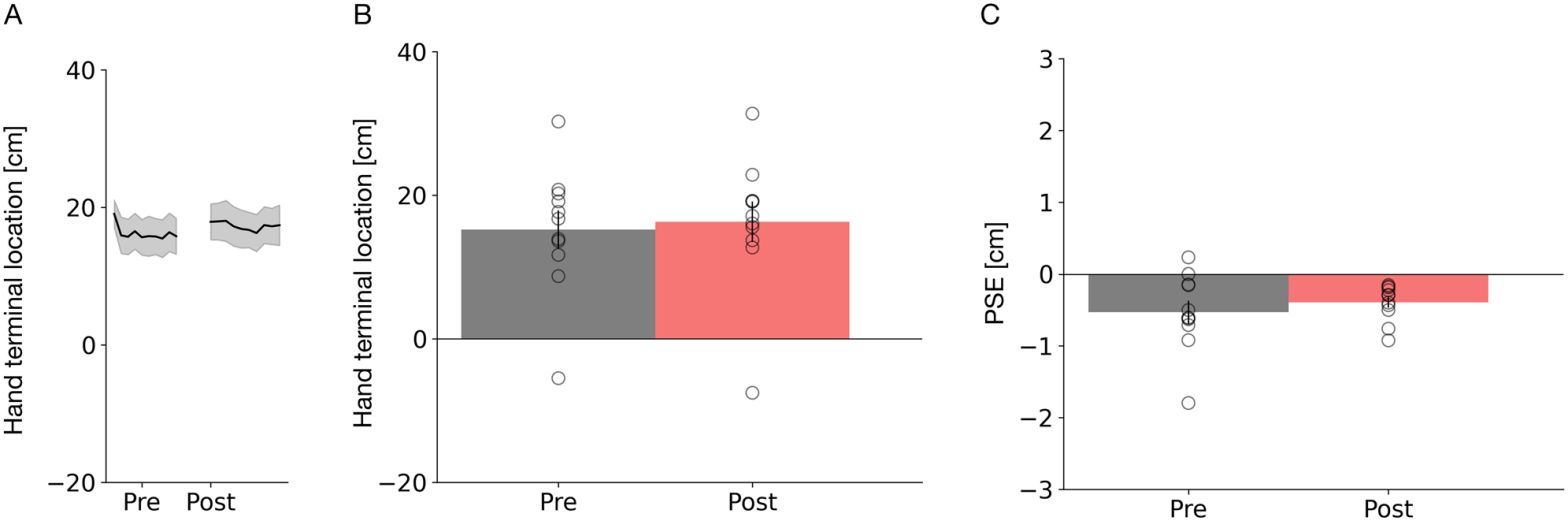
(**A**) Average hand terminal x-components of the blind pointing movement across trials for the “Active pointing without perturbation” experiment. The thick lines represent the mean across all subjects and the shaded area the standard error of the sample mean. (**B**) Average terminal blind pointing locations from before (shown in gray) and after (shown in red) adaptation for Experiment 2. Open circles show single subjects data. Error bars represent the standard of the sample mean. (**C**) Average PSEs of hand localization trials from before (shown in gray) and after (shown in red) adaptation for Experiment 2. PSEs refer to the point where both hands appear at an equal distance relative to the midsagittal plane. Same conventions as in Fig. 5A.

In order to corroborate that the changes in blind pointing and in visual hand localization are related to the adaptive shift and not to other factors, we repeated Experiment 1 with the only exception that the tactile buzz was always presented when the hand reached the target location. In other words, no adaptive shift was applied. Blind pointing results of Experiment 2 are shown in Fig. 5A,B. Under this condition, we found that blind pointing movement amplitudes after adaptation were even slightly higher than before adaptation (β = 1.09, SE = 4.03, t = 2.72, p = 0.007). The visual localization of the right hand (see Fig. 5C), when tested in localization trials, was statistically indistinguishable before and after adaptation (t(10) = − 0.92, p = 0.38). This data indicates that the effects in blind pointing and in visual hand localization, that were observed in Experiment 1, are related to the adaptive shift.

To check if the visual shift observed in Experiment 1 can be observed with 11 participants, we used standard bootstrapping techniques^20^. We randomly selected 11 participants from the pool of 23 participants from Experiment 1. For this sample, we calculated the PSEs of the right hand and checked if the subjects on average localized the right hand further outward than the left hand. We then selected a new random sample containing 11 participants from the pool of 23 participants from Experiment 1 and again checked the average PSE. We repeated these steps 10,000 times. We found that in 96.23% of repetitions the right hand was perceived as further outward than the left hand. This analysis reveals that the effect can also be observed with 11 subjects.

### Experiment 3: active pointing with perturbation—proprioception tested

**Figure 6:**
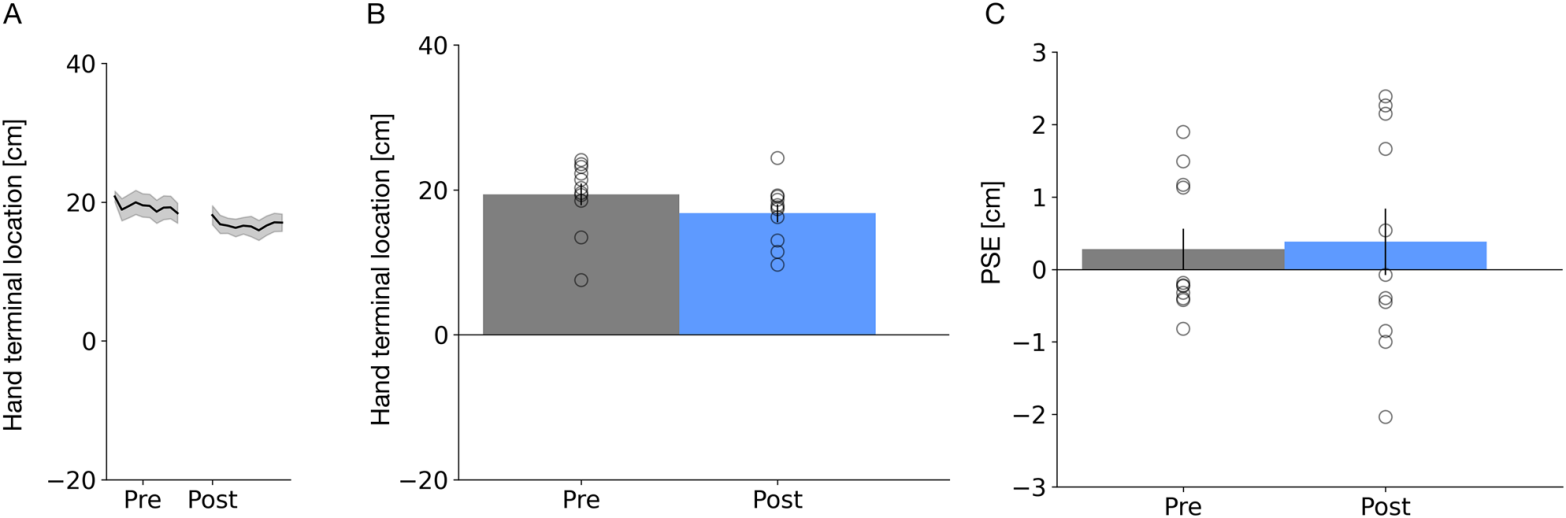
(**A**) Average hand terminal x-components of the blind pointing movement across trials for the “Active pointing with perturbation—proprioception tested” experiment. The thick lines represent the mean across all subjects and the shaded area the standard error of the sample mean. (**B**) Average terminal blind pointing locations from before (shown in gray) and after (shown in red) adaptation for Experiment 3. Open circles show single subjects data. Error bars represent the standard of the sample mean. (**C**) Average PSEs of proprioceptive hand localization trials from before (shown in gray) and after (shown in red) adaptation for Experiment 3. PSEs refer to the point where both hands appear at an equal distance relative to the midsagittal plane. Same conventions as in Fig. 6A.

In Experiment 3, we tested whether our adaptation protocol would also affect the proprioceptively sensed hand location. We repeated the trial structure of Experiment 1, including the adaptive shift in the adaptation trials.In localization trials, subjects compared the proprioceptively determined position of the unseen right hand against a virtual, visual hand presented on the left side. Simultaneously to the presentation of the visual left hand, the right hand was stimulated by a tactile buzz. Blind pointing movements (see Fig. 6A,B), were significantly shortened after adaptation (β = − 0.03, SE = 0.003, t = − 8.32, p < 0.001), thus replicating the finding of Experiment 1. In localization trials, no significant effect of pointing adaptation on the proprioceptive localization of the hand was observed (t(10) = − 0.37, p = 0.72; see Fig. 6C).

### Experiment 4: passive pointing observation

Since in Experiment 1, we found significant outward mislocalization of the right hand, we wondered whether the observation of the visual hand moving might be sufficient to produce a shift in the visual hand location. The pointing adaptation, that we measured in blind pointing trials, might be unrelated to the shift in apparent hand position. To address this question in Experiment 4, participants were tested in a passive version of this paradigm in which, in the adaptation trials, they observed the visual hand moving automatically.

**Figure 7:**
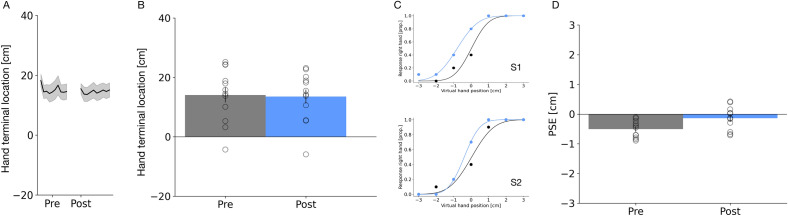
(**A**) Average hand terminal x-components of the blind pointing movement across trials for the “Passive pointing observation” experiment. The thick lines represent the mean across all subjects and the shaded area the standard error of the sample mean. (**B**) Average terminal blind pointing locations from before (shown in gray) and after (shown in red) adaptation for Experiment 4. Open circles show single subjects data. Error bars represent the standard of the sample mean. (**C**) Example psychometric functions for two participants measured in the pre-adaptation (shown in black) and the post-adaptation hand localization trials (shown in blue) in Experiment 4. (**D**) Average PSEs of hand localization trials from before (shown in gray) and after (shown in red) adaptation for Experiment 4. PSEs refer to the point where both hands appear at an equal distance relative to the midsagittal plane. Same conventions as in Fig. 7A.

Figure 7A,B shows average pointing positions before and after adaptation. On average, pointing locations are only slightly shifted inward after adaptation. The respective best fitting LMM only included the factor phase and the random intercept of participants and showed that the final pointing position was shifted closer to the midsagittal plane after repeated exposure to a passively watched hand movement (β = 0.81, SE = 0.34, t = 2.38, p = 0.018). No effect on peak velocities was observed. Pointing peak velocities did not differ between the before and after adaptation (β = 2.72, SE = 2.79, t = 0.98, p = 0.33).

Figure 7C shows example psychometric functions for 2 observers, plotting the proportion of responses “right hand more outward” as a function of virtual hand position. In the condition in which the hand was shown to move as the own hand would move, participants judged the right hand more often to be located more outward after adaptation. Figure 7D shows pre- and post-adaptation results of hand localization trials. Participants perceive the right hand shifted outwards in the normal condition. A one-sample paired *t* test confirmed a significant difference between both conditions (t(10) = − 2.77, p < 0.019). The result of Experiment 4 demonstrates that the mere observation of a hand moving, like the own hand would move, is sufficient to generate a spatial shift in the apparent hand position.

## Discussion

In this study, we created a novel visuomotor adaptation paradigm in which the error signal consisted in an artificial discrepancy between the expected and the actual position of tactile feedback when reaching the goal location. Participants reached to the remembered position of the visual target where they expected to receive tactile feedback. However, in adaptation trials, we shifted the location at which tactile feedback was provided to an earlier point on the movement trajectory. In consequence, participants adapted to the wrong feedback and performed shorter movement trajectories. In principle, participants could have simply stopped their movement as soon as they experienced the tactile impulse. These movements would not be planned to reach the visual target position but to stop upon registration of the tactile stimulus. However, the analysis of the movement peak velocities is not consistent with this interpretation. Peak velocities of the pointing trajectory were reduced after adaptation, indicating that subjects planned a shorter movement. Importantly, the peak velocity was reached before the tactile stimulus occurred. Instead of being stopped because of perceiving the tactile impulse, movements after adaptation must have been planned to reach a closer location.

We found that participants perceived the visual hand to be shifted in the direction opposite to the artificial displacement and opposite to the adaptive shift in pointing. Neither the change in blind pointing, nor the visual shift in perceived hand position can be attributed to a mere drift that occurred due to the prolonged exposure to a dark environment or to other factors unrelated to the adaptive feedback. In a control session (Experiment 2) in which we repeated Experiment 1, except for applying the adaptive shift, we found no change in blind pointing nor in the visual perception of hand position.

A pointing adaptation mechanism driven by sensory prediction errors can be distinguished from one driven by reward prediction errors^20^. In their sensory prediction error adaptation protocol full vision of a cursor, representing the subjects’ movements, and the target was provided. In this condition, subjects altered the predictions about the sensory consequences of their movements, indicated by a shift in proprioceptively sensed hand position. In a second condition, the cursor was seen only late in the movement. In the reward prediction error regime, the cursor was unseen and movement success was only provided by an auditory reward signal. In the reward prediction error condition, although subjects adapted their pointing, no shift in sensory prediction errors, i.e. no displacement of proprioceptive hand position was observed. Our paradigm shares similarities with the reward prediction error protocol with regard to the tactile feedback which represents a reward error signal of movement success. We tested whether our adaptation paradigm affected the proprioceptively sensed hand position and found that this was not the case, in agreement with the earlier findings^22^. It is thus likely that in our protocol adaptation was driven by a reward error signal. The aim of the “Active pointing with perturbation—proprioception tested”—Experiment was to test the possibility that proprioception of the right arm might have shifted during the session and thus might have influenced visual hand localization judgements. To this end, we kept the test trials identical to the visual hand localization, except that no visual right hand was displayed but a tactile buzz was presented on the unseen real hand of the subject. While these test trials represent a direct control of the visual hand localization trials used in our study, general conclusions about proprioception should only be drawn cautiously. Before the start of the test trials, subjects placed their hands by following visually presented cues. Previous studies^17,21–23^ explicitly avoided visual cues by placing hands with a robot manipulandum or through the experimenter in order to circumvent the potentially confounding influence of the visual cues on proprioceptive localization.

In Experiment 4, we found that mere observation of hand movement also is sufficient to evoke adaptive changes in blind pointing and in the spatial visual perception of the hand. Participants watched movies of hand movements while remaining passive. The shift in apparent hand position thus occurs when participants actively move their hands or when they passively observe a hand moving in the head mounted display.

Adaptive changes in pointing without the execution of movements have been reported recently^24^. On a subset of trials, subjects were cued to reach to a target and suddenly cued to withhold the movement. Errors, displayed after trials without movements, were sufficient to induce reaching adaptation. Our data show that adaptation can even occur when no cue to perform an action was issued. However, it is unclear from the present data whether the observation of movements might have automatically triggered movement plans. Several studies have reported that action observation induces brain activation corresponding to the production of these movements^25^. In addition to the mirror neurons that had initially been found in premotor cortex^26^, neurons in primary motor cortex M1 also respond during action observation^27,28^. Two functional types of neurons have been found in M1, those that facilitate action execution and those that suppress it^29^. Activity of facilitative neurons was stronger than that of suppressive neurons during mere observation. This ratio reversed when actions were executed. The central activation of motor cortex during action observation propagated even to the periphery, leading to motor evoked potentials in hand muscles even in the absence of hand movements^30^.

As in all adaptation regimes in which motor errors might be consciously perceived, we cannot exclude that explicit processes contribute to the findings we observe in the present study^31,32^. The amount of mis-directed pointing we observe in the adaptation trials is not fully transferred to the blind-pointing trials. Only the mis-pointing magnitude found in the blind-pointing trials can be considered as true adaptation. In the adaptation trials subjects will stop their movement after receiving the buzz and it cannot be decided if this is due to the feedback or due to adaptation. In blind-pointing trials however, the absence of any feedback about the movement success allows to assess the mis-pointing that is due to adaptation. The intrusion of explicit processes in blind-pointing trials is rather unlikely. Participants were asked after the experiment whether they observed anything unusual in the study and only one subject pointed out the discrepancy between the positions of the visual target and the tactile feedback. We displaced the tactile feedback gradually across trials, thus making it less likely to become consciously detected. Explicit influences on the hand localization task are even less likely, given that the motor error drives pointing closer to the midsagittal plane, whereas the hand is localized further away from the midsagittal plane.

The perception of body parts, especially that of the extremities is tightly linked to the movements they will perform. For instance, visual manipulations of the hand size modifies the movement trajectory that is performed with that hand^33–35^. The appearance of the hand thus constantly updates movement planning. We show that the reverse also holds true: Movement expectation changes where the hand seems to be. If the perception of the hands is very uncertain, sensorimotor predictions might bias spatial estimates of the visual hand location. We created a discrepancy between the seen position of the pointing target and the location at which tactile feedback was received. Our data show that the brain compensates this conflict by shifting the apparent hand position toward the location of the target. Unlike previous visuomotor paradigms, in which either the visual target or a visual cursor, that indicates the hand position^1^, is displaced, we manipulated the tactile feedback. Since the tactile feedback indicated that the hand successfully reached the target, the brain must resolve the spatial discrepancy between the proprioceptively indicated target location and the perception of the hand. Shifts in visual hand location following observation of motor adaptation further confirm that the discrepancy between the final hand location in the pointing trials and the proprioceptively sensed target position is driving the shift in visual hand location. In conclusion, our study shows that visual shifts in hand location follow spatial adaptation of pointing movements when a discrepancy between a predicted and the proprioceptively sensed target location occurs.

## Methods

### Experiment 1: active pointing with perturbation

#### Participants

In Experiment 1, a group of 23 participants took part (10 females, mean age = 25.57 (SD 3.57) years). In Experiment 2, a group of 11 participants took part (8 females, mean age = 24.23 (SD 4.29) years). In Experiment 3, a group of 11 participants took part (9 females, mean age = 26.75 (SD 2.34) years). In Experiment 4, 11 subjects took part (9 females, mean age = 24.4 (SD 5.9) years). All participants were recruited through the Heinrich-Heine University Düsseldorf and received monetary compensation or course credit. All participants had normal or corrected-to-normal visual acuity and reported no history of neurological or movement impairments. Experimental procedures were approved by the local ethics committee of the psychological department of the Heinrich-Heine University Düsseldorf. Written informed consent was obtained before each experiment and the procedure were in accordance with the declaration of Helsinki, except that this study was not pre-registered.

#### Materials

Participants remained seated during the entire experiment. Stimuli were delivered using an HTC Vive Head mounted display (1080 × 1200-pixel resolution per eye, 90 Hz) to immerse them in virtual reality (VR). Participants were provided with a motion controller for each hand. Additionally, participants responded in the experimental tasks by using a pedal for each foot. Motion tracking was accomplished via the SteamVR tracking system. According to previous research the SteamVR tracking system provides a robust tracking of observers' head and hand positions provided tracking loss is prevented^36^. The virtual environment was rendered using a custom-made program created in the Unity game engine, version 2019.1.13f1 (Unity Technologies, San Francisco, U.S.).

#### Procedure

In the virtual environment, participants experienced being in a white room. A white sphere (10 cm in diameter) was placed centrally 10 cm in front of their chest. This sphere served as the start position, where participants had to place their right hand between trials. Participants were instructed to grasp the controller with an outstretched index finger. During the entire experiment a red fixation point (Ø: 1 cm) was presented 25 cm in front of them. Subjects were required to keep their gaze directed at the fixation point. A visual and acoustic error signal appeared when the participants left their predetermined head position. Depending on condition, participants with their physical hand controlled a virtual hand that moved synchronously. The length of the virtual hand was about 26 cm, measured from the tip of the middle finger to the palm base. A virtual arm was attached to the hand in order to increase the impression that the virtual hand belongs to the participants’ body (see Fig. 1A).

Before the experiment started, participants were trained to execute a pointing movement to a blurred target (Ø: 2.5 cm). The target appeared for 100 ms, 40 cm in front of the participant’s head position and 20 cm to the right relative to the midsagittal plane. Participants were instructed to perform a goal-directed movement to the target with a single, uncorrected movement trajectory. When subjects reached the target location with their pointing movement, a buzz was delivered by the motion controller (i.e. a short vibration). Moving the controller back into the start position started the next trial.

Before the experiments, subjects were informed about the trials structure, the task (pointing, hand localization) and the conditions (invisible, flashed, visible). We varied the visibility of the pointing hand in the virtual environment across trials from fully visible to only briefly flashed during the pointing movement and to fully invisible. With this manipulation we aimed to assess the influence of hand visibility on adaptation and in particular on the hand localization. Participants were instructed to keep their gaze directed to the fixation point and how to perform the pointing and the hand localization task. They were not informed about the manipulation and they did not receive any hints about how to interpret the buzz that the controller produced. Before starting the experimental sessions, subjects completed a training session. A training session contained the same amount of trials as the following experimental session.

#### Pre- and post—adaptation blind pointing trials

In 10 blind pointing trials, participants pointed to a flashed visual target without any visual feedback of their hand. Blind pointing trials were performed before and after adaptation. No tactile feedback was provided in blind pointing trials.

#### Adaptation trials

In adaptation trials, participants performed the pointing task. Over the course of the first 10 adaptation trials we gradually shifted the time when the tactile feedback was provided. While in the pre-adaptation trials the tactile feedback occurred when the hand reached the target location, in the adaptation trials the feedback was presented already before the hand reached the target position. The invisible spatial border that determined when the feedback was triggered was shifted away from the visual target in horizontal direction (i.e. closer to the midsagittal plane), 1 cm per trial, until it reached its final distance of 10 cm. In the remaining adaptation trials, this distance of 10 cm was kept constant.

Each participant was tested in three different adaptation conditions that differed in the visual feedback of their hand during adaptation (see Fig. 1D). Either visual feedback of the hand was not available (condition: invisible), was only provided midway through the motion, briefly flashed for 100 ms (condition: flashed) or the hand was visible during the entire adaptation (condition: visible). The conditions were tested in separate sessions and the order was counterbalanced across participants.

#### Re-adaptation trials

Re-adaptation trials were identical to adaptation trials. They were interspersed between hand-localization trials after adaptation, in order to maintain the adapted state.

#### Pre- and post—adaptation hand localization trials

In hand localization trials, participants placed both hands with the controllers on the table in front of them, with outstretched index-fingers pointing on two blue spheres, one on the left and one on the right. The positions of the blue spheres matched the positions at which the virtual hands were flashed. In each trial two virtual pointing hands were flashed for 100 ms simultaneously on each body side (see Fig. 1B). Whereas the hand on the adapted right side was flashed at the real location of the right hand, the left hand was presented equiprobable in one of 7 possible locations that were placed equidistant around the mirror position of the right hand (from − 3 cm to 3 cm, 7 steps of 1 cm). Participants were required to make a two-alternative-forced choice by reporting which hand was closer to the midsagittal plane. Before and after adaptation, 70 trials were measured.

#### Sequence of trials

The basic structure of a session was identical between sessions (see Fig. 1C). To estimate the pre-adaptation performance, participants started with 70 hand localization trials, followed by 10 blind pointing trials. Then, 50 adaptation trials were presented. We set the number of adaptation trials to 50 because in a previous study we observed that this number of trials is sufficient to induce adaptation^37^. To estimate the post-adaptation performance, 10 blind pointing trials were tested. Post-adaptation hand localization trials were intermixed with re-adaptation trials in order to keep a steady state of pointing adaptation. Ten hand localization trials alternated with 10 re-adaptation trials until 70 hand localization trials were completed.

A single session consisted of one hand visibility condition (visible, flashed or invisible) and participants had to complete three sessions in total (see Fig. 1D). The experimental sessions lasted on average 12 min. Between sessions short breaks were included.

### Experiment 2: active pointing without perturbation

Experiment 2 served as control for the adaptation procedure tested in Experiment 1. Since we used a pre/post experimental design, the results might be confounded by a potential fatigue effect or any other process that might build up over the course of the session. To control for this possibility, we conducted an experiment that was identical to Experiment 1, except that no adaptive shift was applied. In other words, the tactile feedback was always applied when the hand reached the position of the target.

### Experiment 3: active pointing with perturbation—proprioception tested

In Experiment 3 we sought to estimate if our adaptation method affects the proprioceptive localization of the hand position. To this end, we repeated the experiment, but changed the hand localization trials.

As in Experiments 1 and 2, in hand localization trials, participants placed both hands with the controllers on the table in front of them, with outstretched index-fingers pointing on two blue spheres, one on the left and one on the right. Only the left virtual pointing hand was flashed for 100 ms simultaneously with a buzz presented on the unseen right hand. Across trials, the left hand was presented equiprobable in one of 7 possible locations that were placed equidistant around the mirror position of the right hand (from − 3 cm to 3 cm, 7 steps of 1 cm). Participants were required to make a two-alternative-forced choice by reporting which was closer to the midsagittal plane, the visually presented left hand or the proprioceptively sensed location of the buzz. Before and after adaptation, 70 trials were measured.

### Experiment 4: passive pointing observation

#### Procedure

The trial structure and the procedures of Experiment 4 were identical to those of Experiment 1, except for the adaptation trials. Participants performed pre- and post-adaptation blind pointing and hand localization trials. In contrast to Experiment 1, however, participants did not perform active pointing movements during adaptation but instead passively viewed computer animated pointing movements of the virtual hand. The replayed trajectories of the hand movement were based on representative pointing movements of a human participant, collected before the experiment. In adaptation trials, participants placed both hands with the controllers in front of them, so that the fingertips of the index fingers matched the position of two blue spheres presented above the table in front of the participants.

### Data analysis

Start of pointing movements was defined as the time when participants left the white sphere that served as the home position. End of the pointing movement was defined as the time when subjects crossed an invisible border 40 cm in front of them. For the analysis, we selected the horizontal pointing x-component from the first sample in which the z-component, that codes the depth dimension, was 40 cm or higher. For the computation of the peak velocities, we used the x and z component of the hand movement tracking data and calculated the euclidean distance between two successive samples as a function of time. To exclude outlier values, we filtered out data in which the euclidean distance of two successive samples was larger than 200 cm/s. In the resulting velocity profiles, the maximum value was selected as the peak velocity.

To test whether pointing speed and final pointing position was influenced by the condition, we fitted a linear mixed model (LMM) using the lme4 package in R. The fixed factors condition (invisible, flashed, visible) and phase (pre, post) were used in addition to the random intercept of participant. We report the results of the best fitting models.

To assess localization errors in the hand localization task, we estimated psychometric functions by fitting a cumulative gaussian to the hand location judgements averaged within each condition and each subject. The resulting psychometric function was used to identify the point of subjective equality (PSE; i.e. the point where both hands appear at an equal distance relative to the midsagittal plane). Mean and SEM of the localization errors pre- and post-adaption were estimated for each condition across subjects.

## Data Availability

The data corresponding to the results shown in the figures are available at https://osf.io/sj4kv/?view_only=0e807a1f64954b42a5a3dfd77a0de546.
